# Development of a Sensitive Induction-Based Magnetic Nanoparticle Biodetection Method

**DOI:** 10.3390/nano8110887

**Published:** 2018-11-01

**Authors:** Jakob Blomgren, Fredrik Ahrentorp, Dag Ilver, Christian Jonasson, Sobhan Sepehri, Alexei Kalaboukhov, Dag Winkler, Teresa Zardán Gómez de la Torre, Maria Strømme, Christer Johansson

**Affiliations:** 1 RISE Acreo, SE-411 33 Göteborg, Sweden; fredrik.ahrentorp@ri.se (F.A.); dag.ilver@ri.se (D.I.); christian.jonasson@ri.se (C.J.); christer.johansson@ri.se (C.J.); 2 Department of Microtechnology and Nanoscience–MC2, Chalmers University of Technology, SE-412 96 Göteborg, Sweden; sobhan.sepehri@chalmers.se (S.S.); alexei.kalaboukhov@chalmers.se (A.K.); dag.winkler@chalmers.se (D.W.); 3 Department of Engineering Sciences, Uppsala University, The Ångström Laboratory, SE-751 21 Uppsala, Sweden; teresa.zardan@angstrom.uu.se (T.Z.G.d.l.T.); maria.stromme@angstrom.uu.se (M.S.)

**Keywords:** magnetic nanoparticles, magnetic biosensing, Brownian relaxation, AC susceptibility, multi-core particles

## Abstract

We developed a novel biodetection method for influenza virus based on AC magnetic susceptibility measurement techniques (the DynoMag induction technique) together with functionalized multi-core magnetic nanoparticles. The sample consisting of an incubated mixture of magnetic nanoparticles and rolling circle amplified DNA coils is injected into a tube by a peristaltic pump. The sample is moved as a plug to the two well-balanced detection coils and the dynamic magnetic moment in each position is read over a range of excitation frequencies. The time for making a complete frequency sweep over the relaxation peak is about 5 minutes (10 Hz–10 kHz with 20 data points). The obtained standard deviation of the magnetic signal at the relaxation frequency (around 100 Hz) is equal to about 10^−5^ (volume susceptibility SI units), which is in the same range obtained with the DynoMag system. The limit of detection with this method is found to be in the range of 1 pM.

## 1. Introduction

Magnetic iron oxide-based nanoparticles (MNPs) are utilized in several biomedical applications, including diagnostics, therapy, actuation, and imaging [1,2,3,4]. In this project, we focus on functionalized iron-oxide based magnetic multi-core particles with a mean particle size of 100 nm as magnetic carriers for the detection of influenza virus. Our aim is to develop a novel, low-cost, portable, rapid, and sensitive detection platform for the influenza virus and pandemic influenza virus. This portable sensing solution aims at shortening the turnaround-time to less than 60 min, while still retaining high analytical sensitivity. The project is focused on detection of influenza; however, the developed methods and instruments are generic and not limited to this specific disease. In the final biodetection system, we will integrate both the fluidic system that handles sample preparation and actuation with the detection method. Alternating current susceptibility (ACS) measurements vs. frequency is used to detect changes in the Brownian relaxation of suspended functionalized MNPs that are induced by volume amplification of the analyte with padlock-probe-ligation and rolling circle amplification (RCA) [5,6,7,8] (in this case cDNA target is used).

In this manuscript, we report on a novel developed ACS measurement system (based on the DynoMag induction technique [7]), where the liquid sample movement into two well-balanced detection coils is based on sample plug-flow driven by a peristaltic pump. Previously we showed that ACS-based bio-detection of MNPs can be made portable with high sensitivity [5]. The detection principle relies on measurements of the changes in the out-of-phase component of the ACS due to the large changes in MNP Brownian relaxation time of suspended oligonucleotide-functionalized MNPs induced by the binding of the MNPs to the large RCA coils. The obtained RCA coils are approximately 1 μm in diameter for RCA amplification time of 60 min.

## 2. Materials and Methods

The magnetic readout is based on frequency-swept ACS measurements of the in- and out-of-phase components of the MNP ensemble from which we extract the Brownian relaxation frequency [7,8,9]. The MNP system used in this study is suspended iron oxide-based streptavidin coated multi-core particles with a median hydrodynamic particle size of 100 nm (BNF, micromod Partikeltechnologie GmbH, Rostock, Germany). For this MNP system, the Brownian relaxation dominates the ACS dynamics in the frequency window of the detection system (up to 10 kHz) [7].

The described magnetic detection method is generic and independent of the target analyte used to create DNA coils. The target analyte used this work was synthetic *Vibrio cholerae* (Biomers, Ulm, Germany), but the achieved measurement results and detection limit are directly applicable to influenza virus. The used MNPs were functionalized as follows: MNPs (10 mg/mL) were washed twice with 1× Wtw buffer (10 mM Tris-HCl, 5 mM EDTA, 0.1% Tween 20, 0.1 M NaCl) using a permanent magnet. The MNPs were thereafter resuspended in 1× Wtw buffer and incubated with 10 µM biotin-conjugated oligonucleotides for 30 min at room temperature. The MNPs were then washed twice with 1× Wtw buffer and re-suspended in Phosphate-buffered saline (PBS) in its original volume. The RCA process was conducted as follows: 5 nM of padlock probes (Biomers, Ulm, Germany) were hybridized and ligated, templated by 15 nM synthetic *Vibrio cholerae* target DNA in a solution consisting of 1× Phi29 buffer, 0.25 mM ATP and 5 mU/μL T4 ligase at 37 °C for 15 min. Circular padlock probes were amplified in 1× Phi29 buffer, 0.1 mM dNTP, 0.12 μg/μL BSA, 4 mU/μL Phi29 polymerase at 37 °C for 1 h, followed by enzymatic inactivation at 65 °C for 5 min (Phi29 buffer, ATP, T4 ligase, dNTP, BSA and Phi29 polymerase from Thermo Fisher Scientific, Waltham, MA, USA). A hybridization mix consisting of 100 mM Tris-HCl (pH 8), 100 mM EDTA, 0.5% Tween-20, 2.5 M NaCl was added to the RCA coil solution. Padlock probe and target sequences can be found in references [5,6,7]. Dilution series were then prepared by stepwise dilution from the stock solution with hybridization buffer. To conjugate the functionalized MNPs to the RCA coils by base pair hybridization, a mixture of MNPs and RCA coils solution was incubated at 55 °C for 20 min. The 0 pM sample, negative control, was prepared by incubating the MNPs with hybridization mix.

A sample volume of 80 µL, consisting of an incubated mixture of MNPs and RCA coils, is injected into a tube by a peristaltic pump. The sample is moved as a plug between the two detection coils and the magnetic moment in each position is read over a range of excitation frequencies. To avoid MNPs sticking to the tube walls, the tube was flushed with a (PBS) solution mixed with 0.05% Tween-20 prior to each measurement. In the DynoMag (RISE Acreo, Göteborg, Sweden) system, we use a paramagnetic powder (Dy_2_O_3_) for ACS calibration. In this new ACS system, since it is not possible to use a solid calibration sample, we instead use a stable MNP system (SHP-20 Carboxyl Iron Oxide Nanoparticles 20 nm from Ocean Nanotech, San Diego, CA, USA), initially measured in the DynoMag system, as a liquid calibration sample.

## 3. Results and Discussions

**Figure 1:**
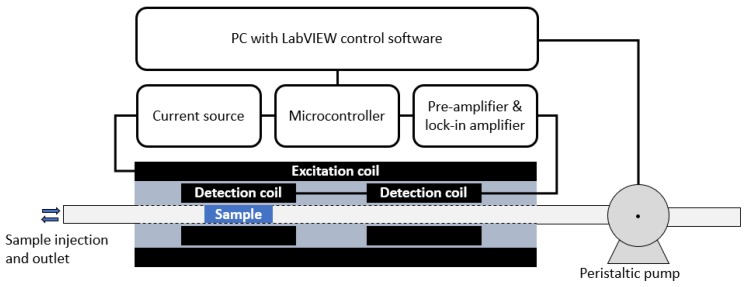
Schematic drawing of the read-out electronics and the coil systems (excitation and detection coils), the sample tube, and the sample. The sample is moved by a peristaltic pump (sample plug flow). The detection coils are coupled in a differential mode (i.e., measuring the difference in magnetic flux between the two coils). The measurement process is controlled by a PC running a LabVIEW program that communicates with the read-out electronics and controls the peristaltic pump.

The ACS measurement system consists of a sample tube with an inner diameter of 3 mm where the sample volume (plug) is positioned in the detection coils with the peristaltic pump system. The baseline centre to centre distance of the two detection coils is 42 mm and the length of each detection coils is 8 mm. A schematic drawing of the read-out electronics, the coil systems, sample tube and sample can be seen in Figure 1. The measurement process is governed by a PC running a LabVIEW control program.

**Figure 2:**
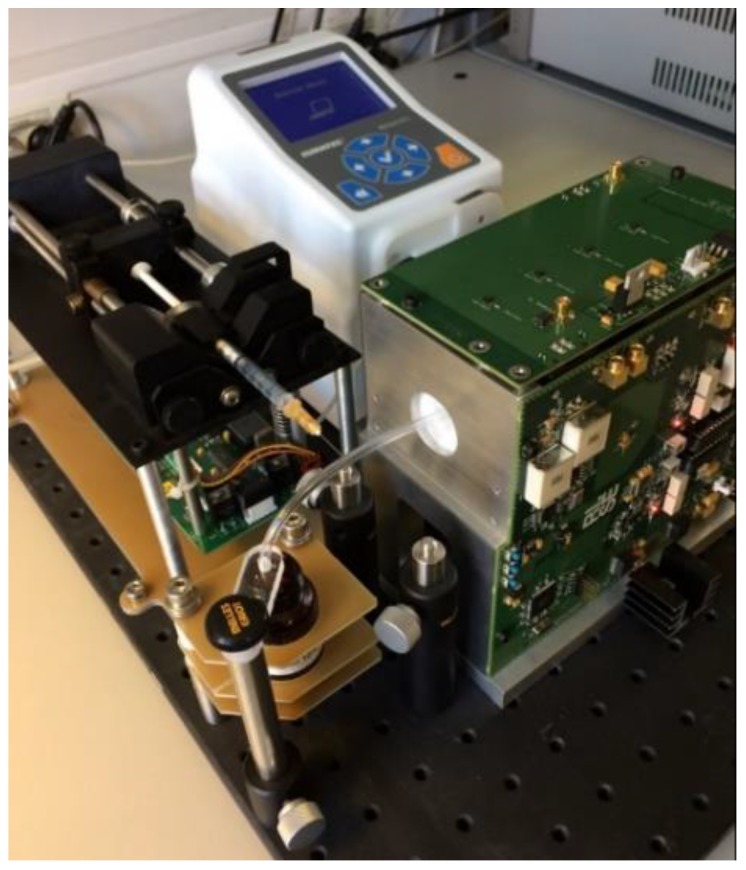
The complete ACS system showing the coil system, electronics and the peristaltic pump system. The peristaltic pump introduces the MNP/RCA sample plug in the sample tube and moves the sample into the detection coil systems. The coil system is behind the electronic parts. A syringe pump is also available as an alternative way to introduce the sample plug into the measurement channel.

A picture of the biosensing ACS system showing the coil systems, electronics (surrounding the coil systems) and the peristaltic pump can be seen in Figure 2. The sample plug is normally pumped in by the peristaltic pump, but Figure 2 also shows a syringe pump, which is used as an alternative method to inject the sample plug into the measurement channel. The needle tip of the syringe is inserted into the sample tube, which allows automatic injection of a well-controlled sample volume. However, the syringe pump has two disadvantages compared to sample injection with the peristatic pump. The first drawback is that a fraction of the sample, approximately 25 µL for the used 1 mL tuberculin syringe with a Luer-slip connection, is still left in the syringe when the plunger is fully inserted. Hence, a fraction of the sample is lost inside the syringe. The second drawback is that the plug can end up on either side of the needle tip or somewhere in between. A possible reason for this apparent randomness in the inserted plug position can be small impurities on the tube wall deposited from prior measurements. With the syringe pump, each sample must be mapped prior to measurement to locate the plug (the mapping process is described below), which adds extra time to each measurement. Therefore, we prefer to use a peristaltic pump for sample injection, which has none of the two drawbacks mentioned above. The end of the sample tube is dipped into the sample container and the desired plug volume is sucked into the tube by the peristaltic pump. The tube is retracted from the sample container before the sample plug is pumped into the coil system.

**Figure 3:**
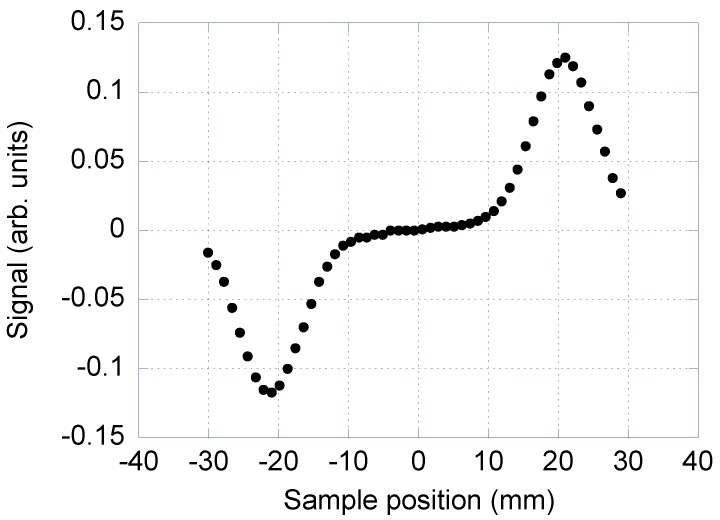
Signal (in-phase component of the AC susceptibility) versus sample position (relative to the coil system). Zero sample position is in the center of the baseline connecting the two detection coils.

To find the optimal position of the sample plug inside the channel, the sample plug is pumped in steps through the detection coil and the signal from the detection coil is recorded at each position. The signal from the measurement system versus the position of the sample relative to the detection coils can be seen in Figure 3. The signal shows a minimum and a maximum when the plug passes the two balanced detection coils, since the detection coils are counter wound. The position of the signal minimum and maximum corresponds to the optimal placement of the sample plug during the measurement.

Measuring both the in-phase and the out-of-phase component of the AC susceptibility vs. frequency [7], we obtained the total time of measurement of five minutes for a complete frequency sweep in the range of 10 Hz–10 kHz (with 20 data points). The reduction in measurement time for our new ACS system as compared to the DynoMag system, is achieved by: (a) reducing the number of measurement frequencies especially at low frequencies, (b) pre-setting the amplifier gain in the electronics (possible since the MNP concentration in the sample is constant and known), and (c) only moving the sample twice during one measurement (to the positions corresponding to the minimum and maximum signal). The obtained standard deviation of the magnetic signal at the relaxation frequency (around 100 Hz) is equal to 10^−5^ (volume susceptibility SI units), which is in the same range obtained with the DynoMag system.

From previous measurements on the reaction yield when forming RCA coils, we know that about 20% of the target DNA is consumed in the RCA reaction [10]. This is taken into account when calculating the RCA coil concentration in this paper. From dilution experiments of the original MNP system [7] and the RCA coil concentration analysis [10], we determined that the mean number of MNPs bounded to each RCA coils is in the range of 10 MNPs per RCA coil.

**Figure 4:**
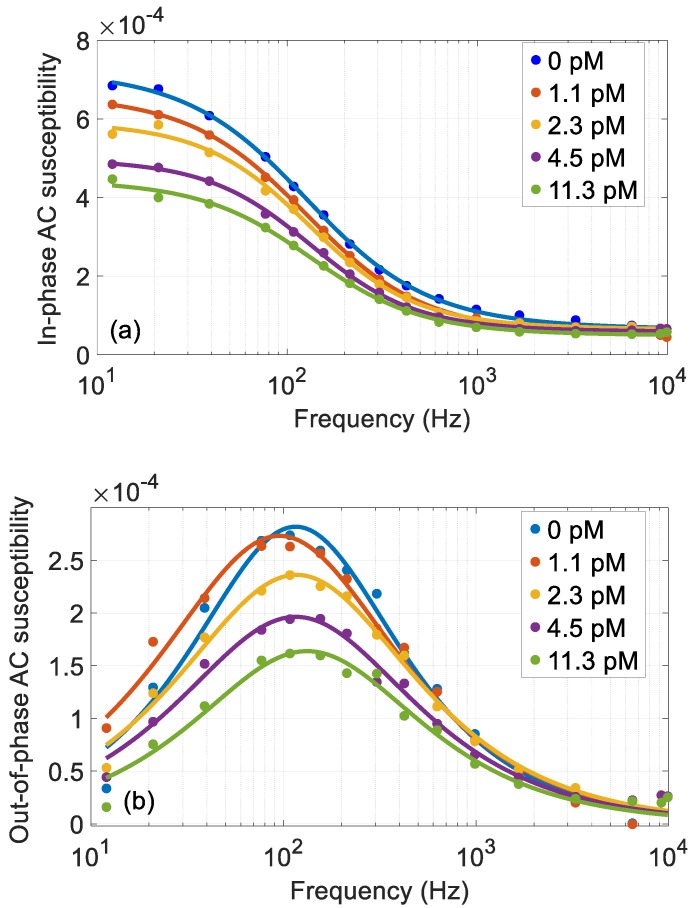
In-phase (**a**) and out-of-phase components (**b**) of the AC susceptibility (volume susceptibility) versus frequency at different RCA concentration as indicated in the figure panels. Measurements are performed at room temperature. The used MNPs have a constant particle concentration of 50 µg/mL for each RCA concentration. The solid lines are from fitting the data to a Cole-Cole function.

The in-phase and out-of-phase components of the ACS vs. frequency for different RCA coil concentrations can be seen in Figure 4. Even if the MNP concentration is low (giving low AC susceptibilities), the ACS response signal is stable and almost free from noise. The assay was run on a dilution series starting from a sample with a high RCA coil concentration. The total number of MNPs (free and bound to the RCA coils) is directly proportional to the in-phase component at high frequencies above the Brownian relaxation [8]. As can be seen from Figure 4a, the in-phase components at 10 kHz coincide for each RCA coil concentration, indicating that we have a constant number of MNPs in all the samples [8]. Reference measurements in the DynoMag system and a high-*T*_C_ superconducting quantum interference device (SQUID) based ACS system have shown a good resemblance between the results.

When the MNPs bind to the µm sized RCA coils, the Brownian relaxation frequency will decrease due to the large increase in the hydrodynamic volume of the MNPs that bind to the RCA coils. The Brownian relaxation frequency for the MNPs that bind to the RCA coils is below 1 Hz and is not visible in the used measurement frequency window. This means that the ACS response from these MNPs more or less disappear from the measurement window of 10 Hz–10 kHz. From Figure 4 we can see the Brownian relaxation (located at about 100 Hz) of the MNPs that are free and not bound to any RCA coil, i.e., the maximum in the out-of-phase AC susceptibility. The data for each RCA coil concentration was fitted using a Cole-Cole function [7]. As can also be seen in Figure 4b, there is a slight increase of the Brownian relaxation frequency when the RCA coil concentration increase, from about 115 Hz for 0 pM to 135 Hz for 11.3 pM RCA coil concentration. This has been observed earlier [7] and is interpreted as an effect of the larger MNPs in the particle ensemble preceding the smaller ones in binding to the RCA coils.

**Figure 5:**
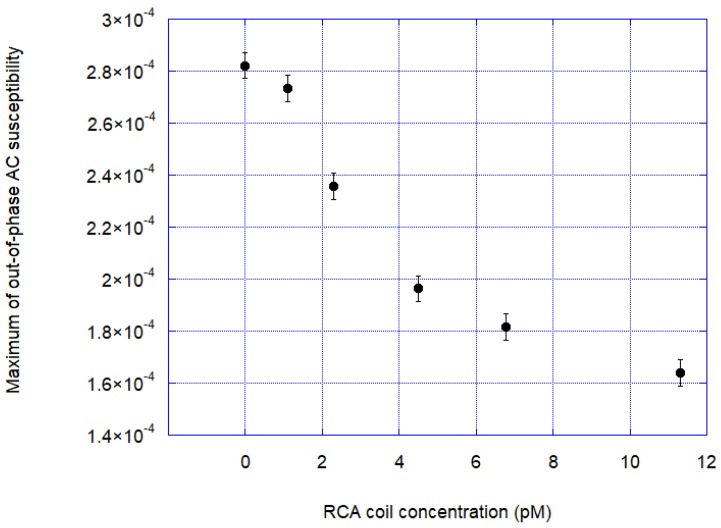
Out-of-phase signal at the Brownian relaxation peak versus RCA coil concentration. Errors bars at each data points are determined from measurements of the signal standard deviation (σ) at the Brownian relaxation frequency (~100 Hz) and is defined by 3σ (about 10^−5^ volume susceptibility SI units).

Figure 5 shows the response curve out-of-phase signal at the Brownian relaxation peak versus RCA coil concentration. From the response curve, we determined an approximate experimental limit of detection (LOD) in the range of 1 pM.

## 4. Conclusions

We have presented results from a new ACS biosensing system that is intended to be used for biodetection of influenza viruses using functionalized MNPs and ACS methods.

With the new ACS system, we obtained a time of measurement that is only five minutes (by sweeping the excitation frequency from 10 Hz to 10 kHz) with almost the same magnetic sensitivity as the DynoMag system (about 10^−5^, volume susceptibility).

We have demonstrated that is possible to use sample plug flow to introduce the MNP sample into the ACS coil system and perform sensitive ACS measurements. This is an important step towards a final biodetection system, where we will integrate both the fluidic system that handles the sample preparation and actuation to the ACS detection system.

The experimental limit of detection (LOD) was found to be in the range of 1 pM.
